# RETRACTED: Kokkinopoulou et al. Associations Between Christian Orthodox Church Fasting and Adherence to the World Cancer Research Fund’s Cancer Prevention Recommendations. *Nutrients* 2022, *14*, 1383

**DOI:** 10.3390/nu16223818

**Published:** 2024-11-07

**Authors:** Anna Kokkinopoulou, Rachel McGowan, Yvonne Brogan, Julie Armstrong, Ioannis Pagkalos, Maria Hassapidou, Anthony Kafatos

**Affiliations:** 1 Department of Preventive Medicine and Nutrition Unit, School of Medicine, University of Crete, 71003 Herakleion, Greece; kafatos@med.uoc.gr; 2 Department of Nutritional Sciences and Dietetics, International Hellenic University, 57400 Thessaloniki, Greece; ipagkalos@ihu.gr (I.P.); mnhas@ihu.gr (M.H.); 3 Department of Occupational Therapy and Human Nutrition and Dietetics, School of Health and Life Sciences, Glasgow Caledonian University, Glasgow G4 0BA, UK; rmcgow203@caledonian.ac.uk (R.M.); yvonne.brogan@gcu.ac.uk (Y.B.); drjuliearmstrong@runbox.com (J.A.)

The *Nutrients* Editorial Office retracts the article “Associations Between Christian Orthodox Church Fasting and Adherence to the World Cancer Research Fund’s Cancer Prevention Recommendations” [1], cited above.

Following publication, concerns were brought to the attention of the publisher regarding a lack of appropriate permission to publish content presented within this article [1].

Adhering to our standard procedure, the Editorial Office and Editorial Board conducted an investigation that confirmed that appropriate permission to publish a portion of the content presented in this publication had not been obtained. As retroactive permission could not be obtained from the intellectual property owner and the content could not be removed without impacting the overall findings, the Editorial Board and the authors have agreed to retract this article [1], as per MDPI’s retraction policy (accessed on 23 October 2024).

This retraction was approved by the Editor-in-Chief of the journal *Nutrients*.

The authors agreed to this retraction.
